# Global Radiotherapy: Current Status and Future Directions—White Paper

**DOI:** 10.1200/GO.21.00029

**Published:** 2021-06-08

**Authors:** May Abdel-Wahab, Soehartati S. Gondhowiardjo, Arthur Accioly Rosa, Yolande Lievens, Noura El-Haj, Jose Alfredo Polo Rubio, Gregorius Ben Prajogi, Herdis Helgadottir, Eduardo Zubizarreta, Ahmed Meghzifene, Varisha Ashraf, Stephen Hahn, Tim Williams, Mary Gospodarowicz

**Affiliations:** ^1^ Division of Human Health, International Atomic Energy Agency, Vienna, Austria; ^2^ Radiotherapy Department, Cipto Mangunkusumo Hospital, Faculty of Medicine Universitas of Indonesia, Jakarta, Indonesia; ^3^ Radiation Oncology, Hospital Portugues, Hospital Sao Rafael, Salvador, Brazil; ^4^ Ghent University Hospital, Ghent University, Ghent, Belgium; ^5^ The University of Texas, MD Anderson Cancer Center, Houston, TX; ^6^ South Florida Proton Therapy Institute, Delray Beach, FL; ^7^ Princess Margaret Cancer Centre, University Health Network, Toronto, ON, Canada

## Abstract

Recognizing the increase in cancer incidence globally and the need for effective cancer control interventions, several organizations, professional bodies, and international institutions have proposed strategies to improve treatment options and reduce mortality along with minimizing overall incidence. Despite these efforts, an estimated 9.6 million deaths in 2018 was attributed to this noncommunicable disease, making it the second leading cause of death worldwide. Left unchecked, this will further increase in scale, with an estimated 29.5 million new cases and 16.3 million deaths occurring worldwide in 2040. Although it is known and generally accepted that cancer services must include radiotherapy, such access is still very limited in many parts of the world, especially in low- and middle-income countries. After thorough review of the current status of radiotherapy including programs worldwide, as well as achievements and challenges at the global level, the International Atomic Energy Agency convened an international group of experts representing various radiation oncology societies to take a closer look into the current status of radiotherapy and provide a road map for future directions in this field. It was concluded that the plethora of global and regional initiatives would benefit further from the existence of a central framework, including an easily accessible repository through which better coordination can be done. Supporting this framework, a practical inventory of competencies needs to be made available on a global level emphasizing the knowledge, skills, and behavior required for a safe, sustainable, and professional practice for various settings. This white paper presents the current status of global radiotherapy and future directions for the community. It forms the basis for an action plan to be developed with professional societies worldwide.

## INTRODUCTION

The WHO states that the highest attainable standard of health is a fundamental right of every human being.^1^ Factors such as availability, accessibility, acceptability, and quality are fundamental to obtain the highest attainable standard of health.^2^ Although there is mounting evidence that good health care can benefit the gross domestic product, radiotherapy is still seen as an expense rather than a cost-effective investment in most countries. The Lancet Commissions offer valuable information on many opportunities offered by good access to health services^3^ and take into consideration the Global Burden of Disease and health challenges faced by the global community.

**CONTEXT**

**Key Objective**
To encourage a unified approach to addressing many of the radiotherapy challenges and avoid wasted resources and duplication of efforts. Fifty percent of all patients with cancer worldwide require radiotherapy, and access to this essential treatment is very heterogeneous. Many organizations and institutions are working to identify and address gaps in resources and to harmonize and support education and training of personnel.
**Knowledge Generated**
Gaps in access to radiotherapy will be presented. In addition, global and regional initiatives that support education and training, assessment of radiotherapy availability, radiotherapy research, and future directions will be highlighted.
**Relevance**
Knowledge of the current status and future directions of global radiotherapy and the many ongoing initiatives will form a basis for a unified action plan and framework to streamline collaborative efforts and lead to a more efficient use of resources.

As countries improve the outcomes in infections and cardiovascular diseases, cancer, a major noncommunicable disease, continues to emerge as a major global health concern. An estimated 9.6 million deaths in 2018 was attributed to cancer, making it the second leading cause of death worldwide. Left unchecked, the problem will only increase in scale, with an estimated 29.5 million new cases and 16.3 million deaths occurring worldwide in 2040.^4^ Cancer requires complex interventions in prevention, diagnostic, therapeutic, and palliative and supportive care services. The availability of comprehensive, responsive, high-quality services for cancer would automatically address the many needs of an effective health care system. Support of cancer care can act as an anchor for the health system to cover other cross cutting areas. Unfortunately, many countries with increasing burden of cancer possess very limited capacity to deal with this disease because of lack of infrastructure, human resources, and access to various components of cancer management.^5^

To review the current status of radiotherapy within the global cancer control framework, the International Atomic Energy Agency (IAEA) convened a meeting of experts in November 2018. The purpose was also to identify opportunities for joint activities in support to radiation oncology worldwide. The meeting also reviewed opportunities for outreach, advocacy, and communication strategies to support funding initiatives for global radiotherapy. Finally, recent advances and future research directions in radiation oncology were also reviewed. The aim of this paper is to summarize the discussions and to present the future directions agreed during the meeting.

There is ample evidence that roughly half of all patients diagnosed with cancer require at least one course of radiotherapy during their disease history^3,6,7^ and reaching 87% in breast cancer.^8^ Radiotherapy is a very cost-effective treatment and is a critical component of effective cancer services worldwide.^3^

Globally, cancer consumes around 5% of the national health expenditure and radiotherapy expenditure also constitutes around 5% of the total cancer cost.^9^ Even with all these numbers and evidence, the intrinsic complexity and regulations complicate the investment in the field.

Access to radiotherapy worldwide is very heterogeneous, whereby the socioeconomic conditions of a country often correlate with the available resources.^10,11^ The current median and range of density of radiotherapy machines per million population are 5.1 in high-income countries (HICs) (range, 0.4-11.6) and 0 in low-income countries (range, 0-0.4).^12^ The significant investment required for setting up a radiotherapy program and the continuous operational and maintenance needs make it challenging for low- and middle-income countries (LMICs) to initiate and maintain sustainable growth in radiotherapy access on a national level, especially in the face of competing public health and other development priorities.^3^ This picture holds true even in HIC where greater health care funding is available.^3,6^ Nevertheless, even in countries with more machines, challenges remain regarding the appropriate utilization of radiotherapy, because of difficulties in technology implementation, issues related to safety and quality control, and the lack of continuing education and training of professionals.

## GLOBAL COLLABORATION IN RADIOTHERAPY

There is a huge discrepancy in the accessibility to cancer services globally, as well as availability of services, affordability of care, and awareness of potential benefits of modern cancer care. These barriers must be addressed one by one to improve population-based outcomes.

There are many institutions and professional bodies engaged in global health, each with a specific mandate and mission objectives. In response to the world's growing cancer crisis, many of these actors are joining efforts to improve cooperation and coordinate their efforts to maximize the use of available resources. The WHO and the IAEA have been involved in advocacy and numerous actions to improve cancer control.^13,14^ The WHO has adopted multiple resolutions calling for improved access to palliative care, surgery, essential medicines, and overall cancer control. Similarly, the IAEA has been engaged in educating, advising, and supporting countries to provide safe nuclear-based technologies for health. In this context, the IAEA has been working on improving access to safe and efficient radiotherapy, diagnostic imaging, and nuclear medicine services. To attain this goal, the IAEA provides guidelines and supports procurement for new and existing facilities. Furthermore, the IAEA manages the web-based international directory of radiotherapy centers, which is the only database on worldwide radiotherapy resources.^15^

The recently published third edition of the Disease Control Priorities (DCP3)^16^ proposed a series of best buys in cancer control. Radiotherapy is now included in most comprehensive cancer control plans. In addition, awareness of the various milestones that need to be achieved to establish nuclear medicine, diagnostic imaging, and radiotherapy services is essential.^17^

The International Cancer Control Partnership portal collates available national cancer plans and guidelines for implementing comprehensive cancer services. The Union for International Cancer Control has a new initiative,^18^ Treatment for All, that calls for creating partnerships to improve access to cancer treatments. Although prevention is an essential component of cancer control, not all cancers can be prevented, and many can be effectively treated. The new initiative created by Union for International Cancer Control City Cancer Challenge (CCan) works in cities worldwide with more than one million people in LMICs to improve access to cancer care.^19^ The CCan effort is supported by a multisectoral group of stakeholders including civil society, academia, industry, and United Nations (UN) agencies, including the IAEA that actively supports radiotherapy, radiodiagnosis, and nuclear medicine portion of the CCan initiative.

## AVAILABILITY AND HOW TO ASSESS NEEDS IN RADIOTHERAPY

Assessing the needs and gaps in radiotherapy at a country or regional level is the first step for adequate planning of the processes, human resources, and infrastructure. Data-driven health care planning presents not only tremendous opportunities but also many challenges in data collection and evaluation.^20^

One example is the research done by the Health Economics in Radiation Oncology Project of the European Society for Radiotherapy and Oncology (ESTRO-HERO). This project demonstrated a six-fold variation in the number of mega voltage machines per million population, 15-fold variation in the number of radiation oncologists per million population, and 20-fold variation in the number of medical physicists per million population.^21,22^ Interpreting personnel data across countries is, however, complicated by the fact that within each country, the radiation oncology professionals take up different professional roles and responsibilities.^23^ In addition, differences in resource availability can be explained by the variation in cancer incidence and socioeconomic considerations, the stage in technology adoption, and related treatment complexity, resulting in considerable variations in courses delivered per professional and per mega voltage unit per year.^24^ The result is that at least one of four patients with cancer does not get access to evidence-based radiotherapy.^6^ Besides socioeconomic aspects, factors such as comorbidity and older age; physician bias, with specialists tending to recommend their own therapies; and geographical access, determined by the distance to the hospital, all play a role in the variation in accessibility.

**FIG 1:**
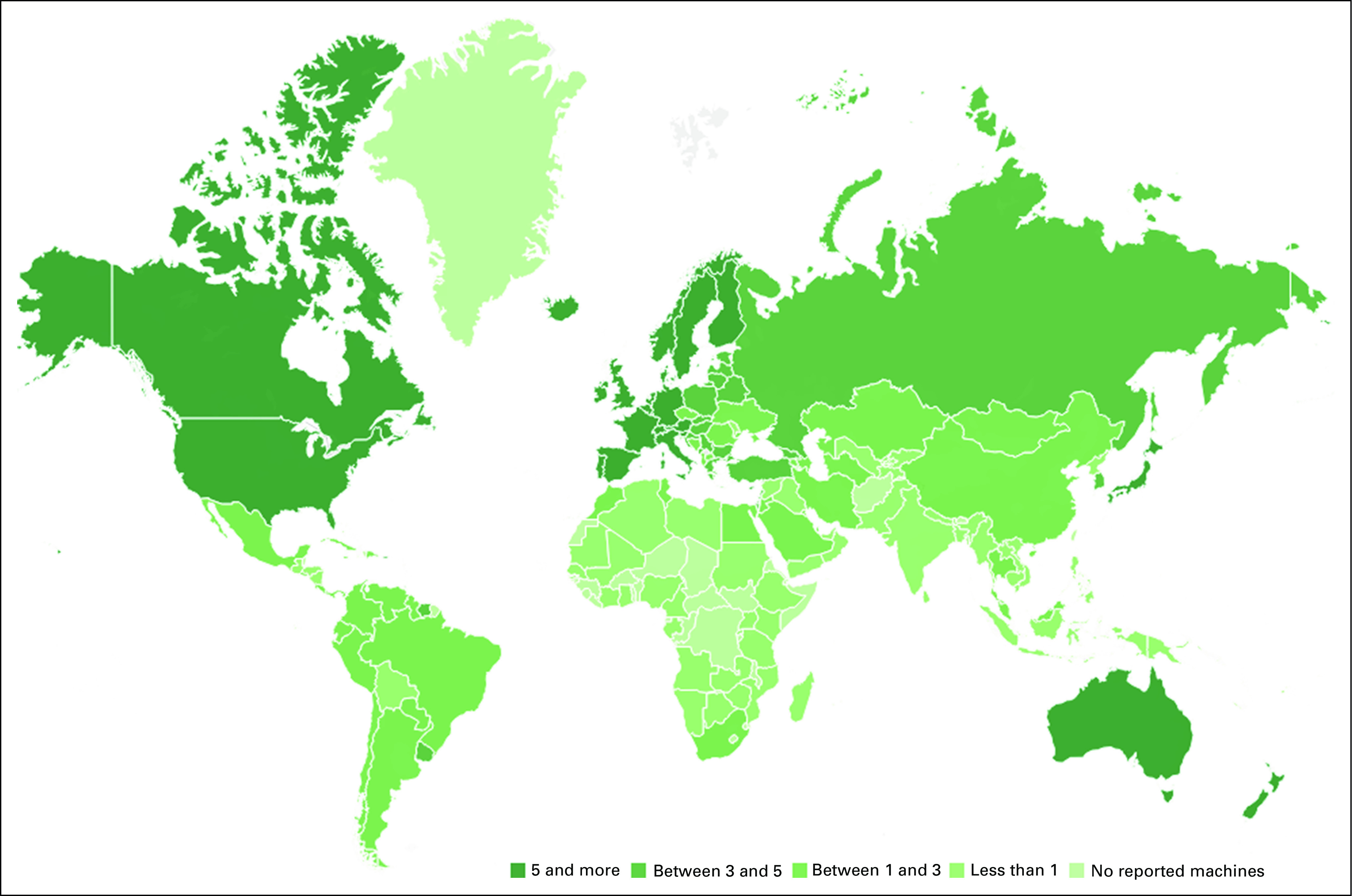
Access to radiotherapy worldwide per million population.

At the global level, the situation is challenging (Fig 1; Tables 1 and 2). This is especially seen in LMICs. By 2040, 67% of annual cancer cases will be in LMIC and there is no adequate resource mobilization to tackle this future challenge.^12^ The Lancet Commission analyzed the burden and demand of radiotherapy worldwide and demonstrated the stark inequities in its availability globally.^3^ The Lancet Oncology Commission report on radiotherapy quantified the gap in access to radiotherapy and the cost of closing this gap. It presented an investment case to support scale-up of radiotherapy worldwide and the potential benefit to the gross domestic product when proper access to radiotherapy is available and used in curative cancer care. Their findings concluded that the potential to save nearly one million lives per year by 2035 through optimal access to radiotherapy would lead to a net macroeconomic benefit of up to $365 billion in US dollars (USD) over the 20-year scale-up period. The Global Impact of Radiotherapy in Oncology^25^ project of the ESTRO aims to continue the effort of the Global Task Force on Radiotherapy for Cancer Control (GTFRCC) in promoting the awareness of the benefits of radiotherapy worldwide.

**TABLE 1:**
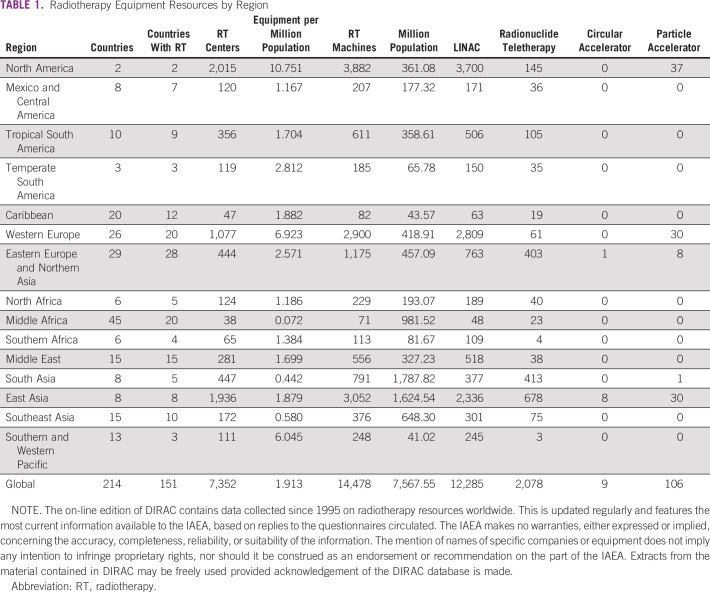
Radiotherapy Equipment Resources by Region

**TABLE 2:**
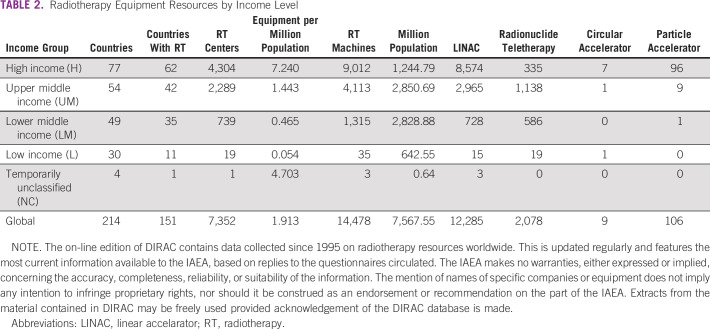
Radiotherapy Equipment Resources by Income Level

For example, if we consider the Asia Pacific region with its high population density, it poses the largest challenge regarding the absolute numbers of capital resources to invest in and of human resources to train to ascertain full radiotherapy coverage. Africa, conversely, with 27 countries lacking any radiotherapy whatsoever, is confronted with the challenge of building radiotherapy resources, with considerable incremental steps to be taken to cover for radiotherapy needs.^11,13,26^

Actual recommendations on the number of radiotherapy resources needed typically represent a snapshot in time and insufficiently account for the rapid evolution in radiation therapy indications, techniques, and fractionation schedules.^23^ Several costing models are available, based either on the use of time-driven activity-based costing methods^3,27^ or cancer type–specific costing model.^28^

Radiotherapy is an affordable treatment. However, radiotherapy only consumes roughly 5% of the total cancer care budget and about 0%-5% of the total health care budget, whereas the highest proportion of cancer care costs is typically related to drugs and in-hospital stays.^29,30^

The GTFRCC calculated that global investments in radiotherapy needed over 20 years to close the gap is estimated to be about $184 billion USD. The cost would be halved in case maximal efficiency could be ascertained. The benefits of such investment are staggering: by 2035, optimal radiotherapy would allow us to save one million lives annually. The GTFRCC calculations thus demonstrated a clearly positive return on investment, with up to a $6 USD return for each dollar invested.^3^ Despite the strength of such data, there remains a need for further evidence on the value of radiotherapy, defined as “health outcomes that matter to patients in relation to the costs of delivering these outcomes.”^31^ Existing value tools developed for oncology mostly focus on the value of oncology drugs and are not simply transferable to the context of nonsystemic treatment strategies. This is due to the different nature of innovations in surgery and radiotherapy, as well as the outcomes obtained and the evidence needed to evaluate it.^32^

Increased effort is needed to work with the device manufacturers to bring down the acquisition costs of the treatment machines and allow the most efficient use of resources. More standardization into the operation of the machines and improved interconnectivity is also needed.^33^ Guidance regarding prerequisites for developing new technology should furthermore be made available.^34^

## EDUCATION AND TRAINING OF PROFESSIONALS IN RADIOTHERAPY

In addition to equipment and facilities, the availability of radiotherapy professionals is crucial for ensuring a sustainable and functional radiotherapy program. A shortage of trained professionals is a serious hurdle to overcome in making radiotherapy accessible to patients with cancer.^10^ Addressing this issue requires significant time and effort to develop and implement strategies in education and training that correspond to the unique realities and challenges faced by each country. These educational initiatives, at present, tend to be country- or region-specific and somewhat isolated from each other because of differences in standards and regulations governing medical practice and education.^35^

Trained professionals are key for the sustainable growth of radiotherapy and should be a strategic consideration in any National Cancer Control plan. Not only the number of professionals but also, more importantly, the quality of professional training must be considered.^36^ Supporting initial education and training of radiotherapy professionals, such as medical physicists, radiation therapists, and radiation oncologists, as well as continuing education and training of previously trained professionals to update or expand their knowledge and skills is a priority.

Considerations of the specific needs of individual countries and regions are essential for improving outcomes. The global coordination by organizations such as the IAEA with their support of long-term and short-term fellowships, education and training workshops, and virtual education platforms continue to be instrumental in supporting this cause.^13^

Shortages of radiotherapy professionals have been reported. For example, in Asia, these shortages are seen in both LMICs such as Sri Lanka and Bangladesh, as well as high- and middle-income countries such as Japan and South Korea. In a recent survey by the Federation of Asian Organizations for Radiation Oncology member countries,^37^ only 54% of the region's need for radiation oncologists have been fulfilled, with most countries producing < 10 new radiation oncologists per year while facing a shortage of more than 200 machines. At their current capacity, training capacity becomes an important bottleneck in achieving the important milestone of 1 megavoltage unit per million population. A similar picture exists with medical physicists and radiation therapists with a lack of sufficient training programs for radiotherapy medical physicists and radiation therapists in many countries. A similar situation has been reported in Africa where only a small proportion of countries have training programs in place for professions related to radiotherapy.^35^

Producing the required number of trained personnel is already a significant challenge today, and the constant increase in demand will only add to this problem. A training program can only support a limited number of trainees at any given time, partially depending on the number of available teaching staff. The limited capacity for training is further reduced by the high patient workloads of the existing professionals, leaving less time for teaching. Acceleration of the education and training of new radiation oncology professionals must not lag too far behind the rate of increase of cancer incidence. Such a delay would make training of a sufficient number of trained professionals even more difficult if not impossible. In addition, the duration of personnel training and adequacy of the training institution are other areas that can be inconsistent and present challenges to adequate human resources.

Furthermore, it is important to note that the relative surplus capacity in one country does not necessarily contribute to reducing the shortage in other countries, owing to the restricted nature of the movement of health care professionals across national borders. Mutual recognition is helpful to allow flow of health care professionals, but is not without its caveats, including preferential movements in only one direction causing a phenomenon known as brain drain, which causes even more inequality on a regional or global level.^38^ Therefore, it is more realistic to aim for allowing the utilization of excess training capacity in one country to improve the capacity and/or quality of the education and training of radiotherapy professionals in other countries.

With a diverse global education network, it is immediately apparent that no single system is accepted on a global level for the education and training of radiotherapy professionals. The diversity of systems, models, and requirements for education and training of radiation oncology professionals across the globe reflects the different needs of each country.^35^ It is possible, however, to identify common standards, requirements, and competencies across the different systems. Best practices have also been shared and adapted among systems, and it has become increasingly common for system-neutral educational initiatives to be made available and used globally.^39,40^ The IAEA syllabi for radiation oncologists are good starting points for the development of a minimum standard.^41^

**FIG 2:**
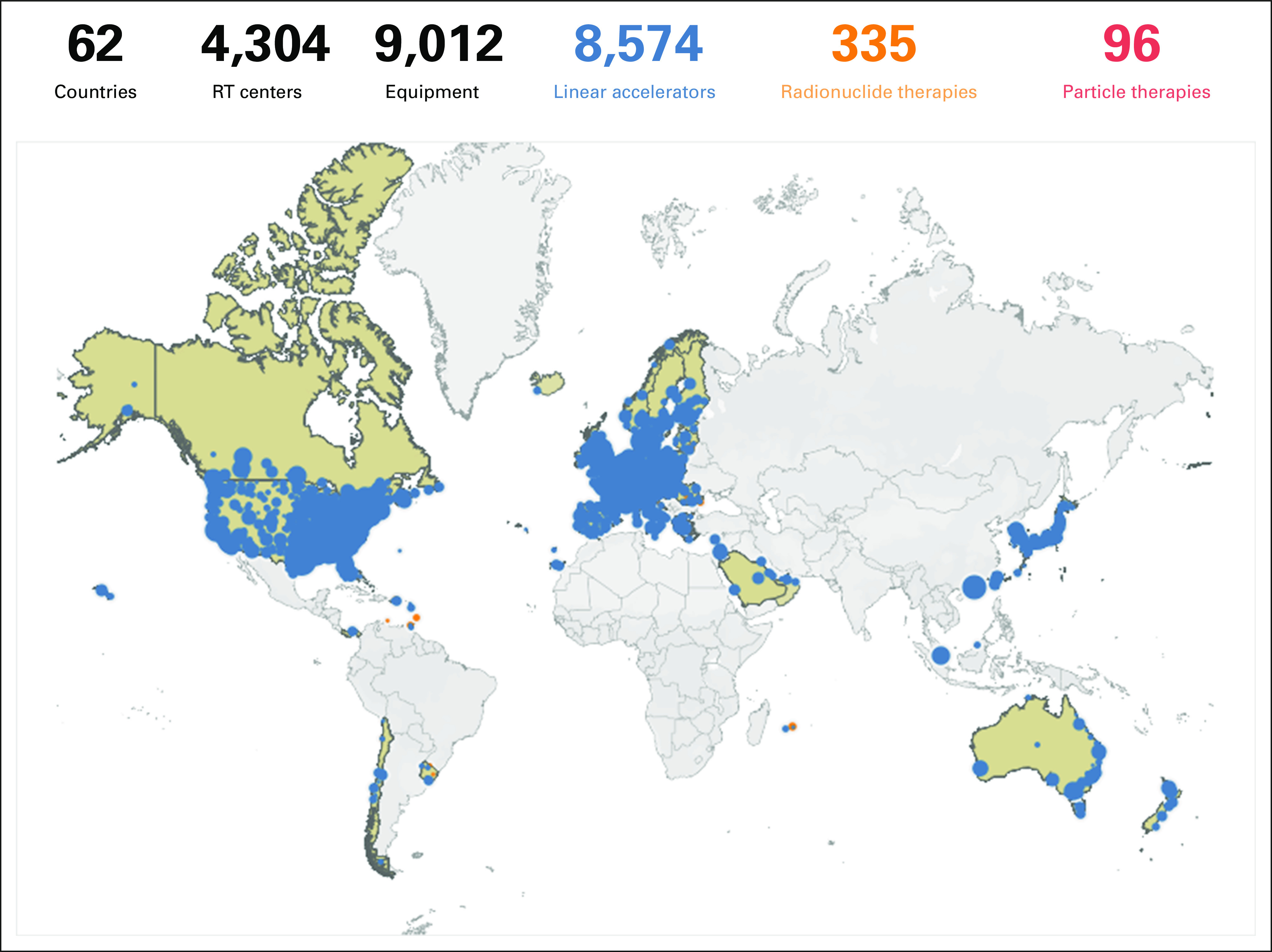
Distribution of radiotherapy centers in high-income countries.

**FIG 3:**
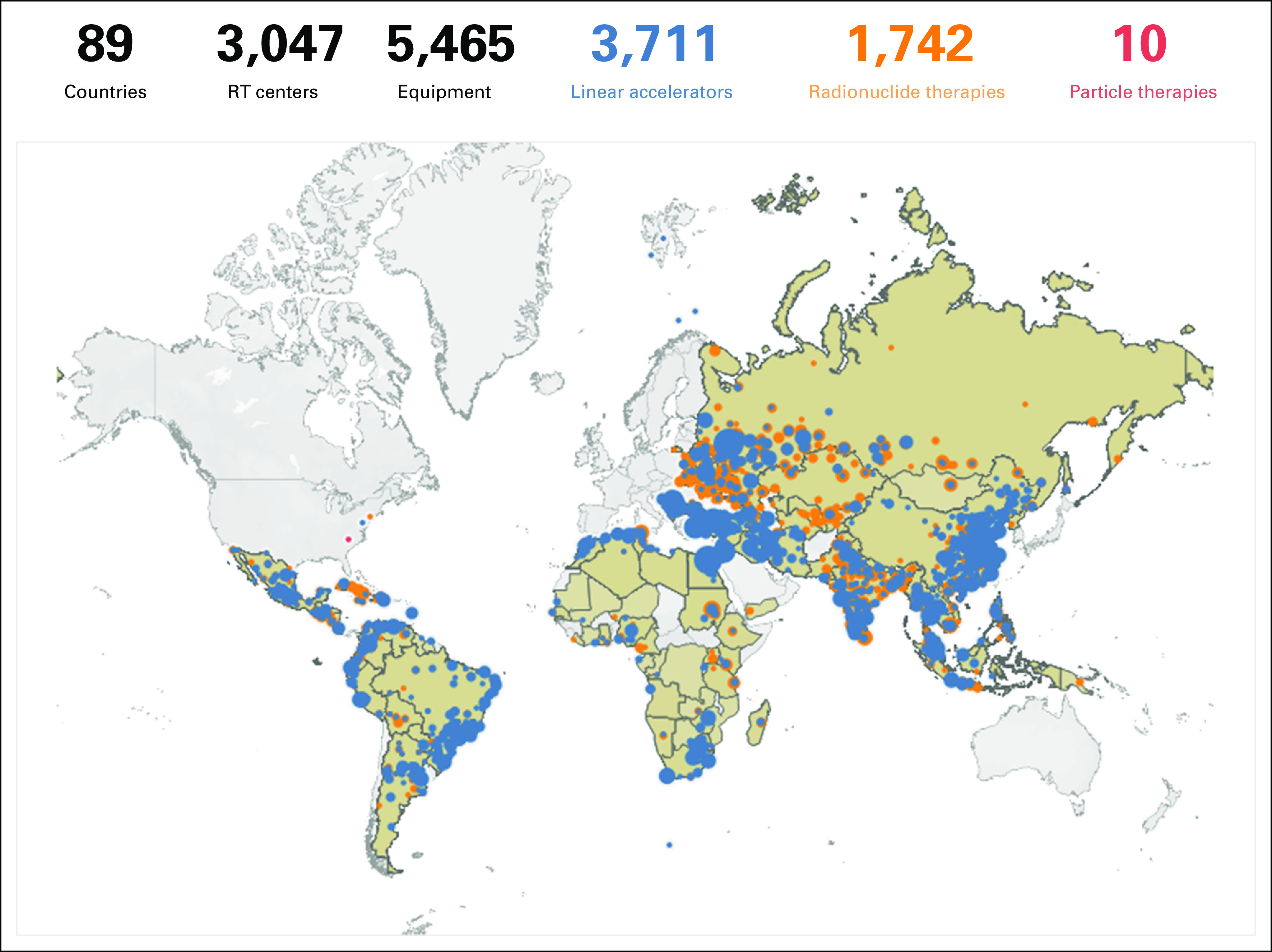
Distribution of radiotherapy centers in low- and middle-income countries.

**FIG 4:**
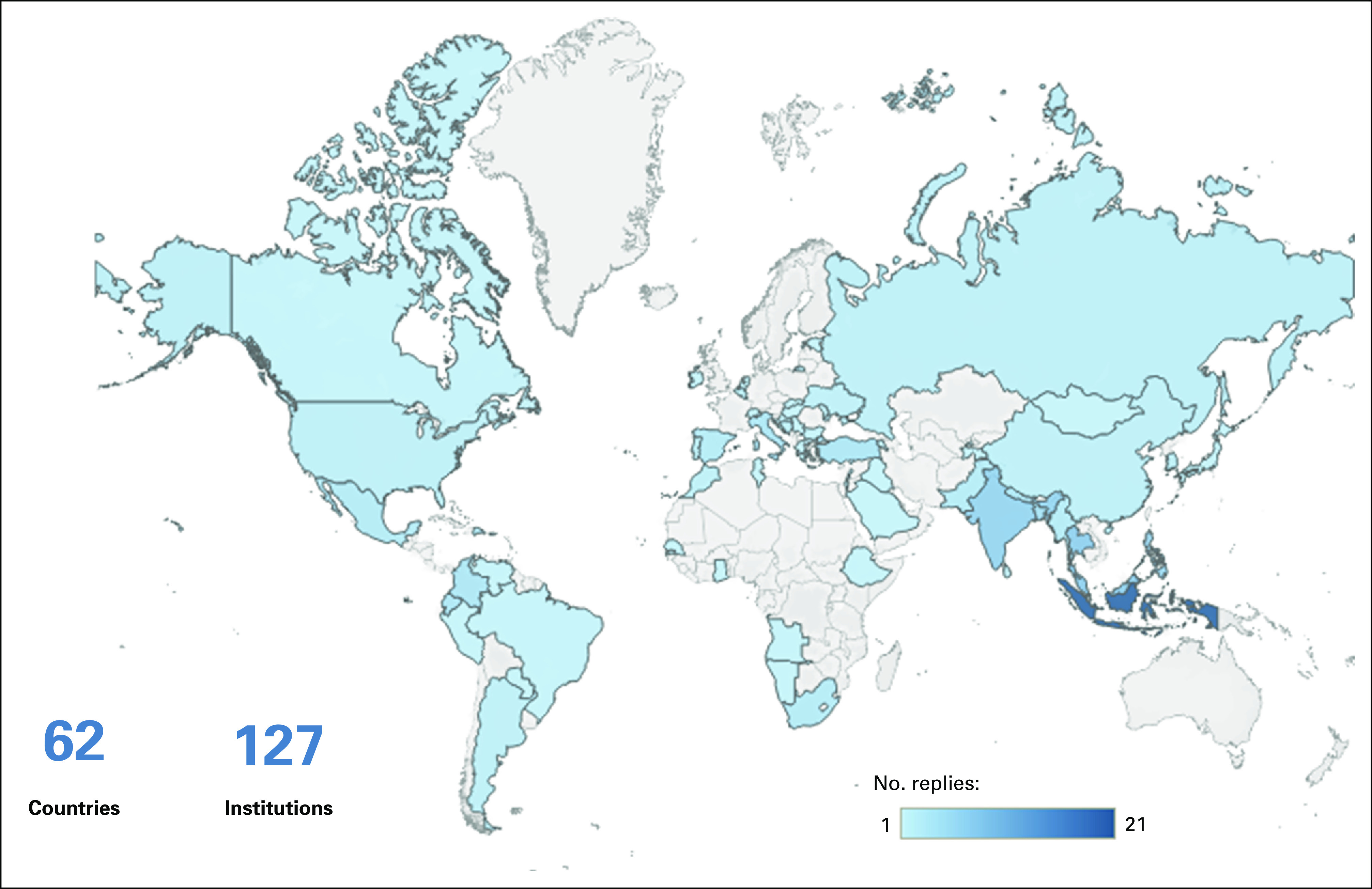
Countries that replied to the survey.

**FIG 5:**
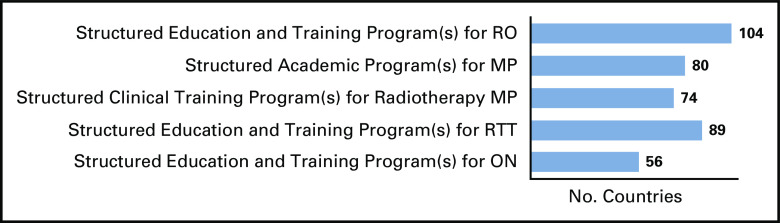
IRIS^42^ survey results based on countries who responded to have educational and training program(s) for RO. IRIS, International Research Integration System; MP, medical physicists; ON, oncology nurses; RO, radiation oncologists; RTT, radiation therapists.

The plethora of global and regional initiatives, however, would benefit further from strengthening the information systems and the existence of a central framework, an easily accessible repository through which resources can be accessed and inquiries can be made. Using the IRIS^42^ platform, the IAEA is currently developing a database of educational resources in radiotherapy at a worldwide level. Through a series of surveys, formerly unknown training facilities and resources have been identified, which brings new opportunities for the education and training of radiotherapy professionals (Figs 2–5 and Data Supplement).

## THE IMPORTANCE OF RESEARCH IN GLOBAL RADIOTHERAPY

Research is a key pillar for the long-term improvement of cancer control, along with clinical and education or training activities, ensuring progress and scientifically based management in radiotherapy. However, the nature and implementation of research activities may vary according to the resources available locally and the interaction with the rest of the research network in the country. Research units create an environment of excellence and leadership and contribute to medical scientific knowledge in the field of radiation oncology, all aiming at improving quality of life and cancer outcomes.

**FIG 6:**
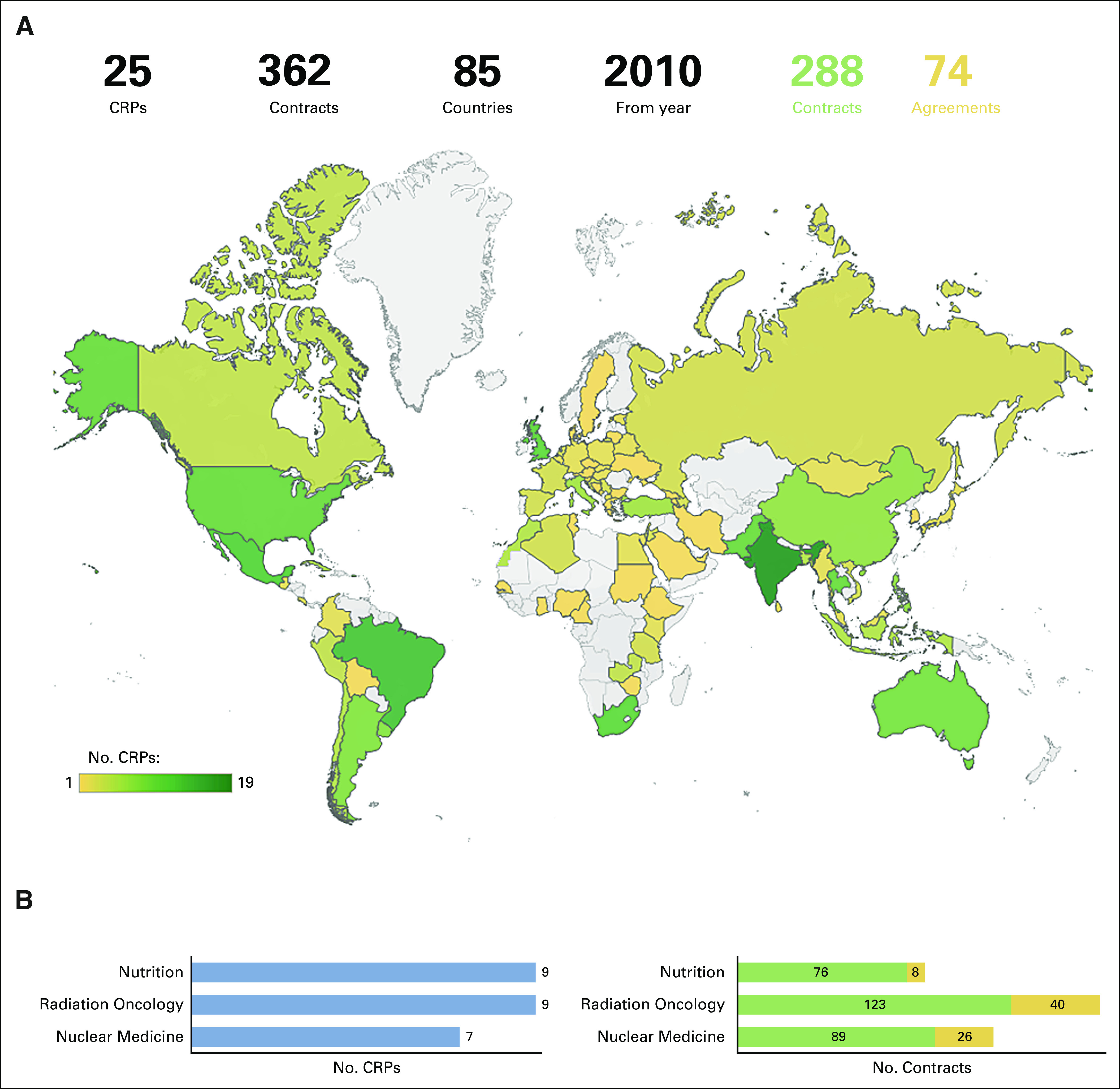
(A) Overview of the distribution of IAEA's CRPs and contracts worldwide. (B) Distribution of CRPs and contracts worldwide by specialty (updated on February 09, 2021; 13:46:45). CRPs, coordinated research projects; IAEA, International Atomic Energy Agency.

International multi-institutional global research, such as the IAEA's Coordinated Research Program (Fig 6), which includes clinical trials, has many unique benefits.^14,43^ This type of research has the added value of introducing radiation oncology professionals in LMICs to new clinical research activities and evidence-based medicine. In addition, it facilitates collaboration with researchers and investigators in HICs. The results can minimize site selection bias. For example, the results of research from HIC institutions alone may not translate directly when used in LMIC. Participation from a wider variety of countries and clinical settings is more likely to produce realistic results transferrable to the LMIC setting.

In addition, implementation research is very important in radiation oncology to address the knowledge gap between evidence-based interventions and their delivery to community practice, particularly in LMICs. Research is needed to identify the complexities in health systems and cost evaluation of interventions^27,44^ and to define sustainability strategies.^45^

Educational research, such as coordinated research activities, is imperative in supporting research worldwide as it can bring various benefits to the participating countries. These benefits can be in the form of presenting opportunities for scientists and institutions to conduct research that would otherwise not be possible. Furthermore, coordinated research activities provide access to specialized and experienced researchers in various fields and to research networks worldwide, which can lead to resource sparing, training in the use of new technology, and overall support to future research activities in the country.^13^

## ADVOCACY AND OUTREACH IN GLOBAL RADIOTHERAPY

To date, there are several ongoing initiatives trying to tackle this precise question, involving various stakeholders including international bodies such as UN, government institutions, national and regional medical societies, and nongovernmental organizations, all of whom share a common objective, improving radiotherapy and making it accessible to all. There are many examples of past or ongoing initiatives.^3,46–49^ Among the work and initiatives conducted by these various stakeholders, international organizations, and medical entities and institutions, there is often overlap in the work, recommendations, and support provided to countries. Nonetheless, there is not always a unified approach to addressing many of the challenges faced today in raising awareness and understanding regarding the role and importance of radiotherapy and increasing its accessibility. For this reason, issues are often not addressed comprehensively on a global scale but rather nationally or regionally, meaning that the results are not always reproducible and applicable worldwide.

## FUTURE DIRECTIONS FOR GLOBAL RADIOTHERAPY

During the meeting, future directions for the global radiotherapy community were also discussed. The following is a summary of these discussions.

The advent of new communication technologies made the creation and distribution of information easier than ever. There is a need to generate new global public information and discourse in all areas of the society, including health care in general and radiotherapy in particular. The exponential growth of information highlighted the need for globally accepted guidelines, practices, and partnerships. Unified and coordinated guidance can prevent duplication of efforts and promote rapid dissemination of best practices. The IAEA has produced many publications with guidelines on how to establish a new radiotherapy department and guide quality control programs, safe practices in radiotherapy, and others.^50^

Furthermore, tools and technical packages are needed on readiness assessment for radiotherapy, adaptation of guidelines for local adoption, implementation of new technologies, and others. Useful tools for countries must be developed assuming progressive growth of facilities and programs over time. However, a note of caution regarding the ethical implications is needed, to avoid the adoption by countries of inferior treatments.

**FIG 7:**
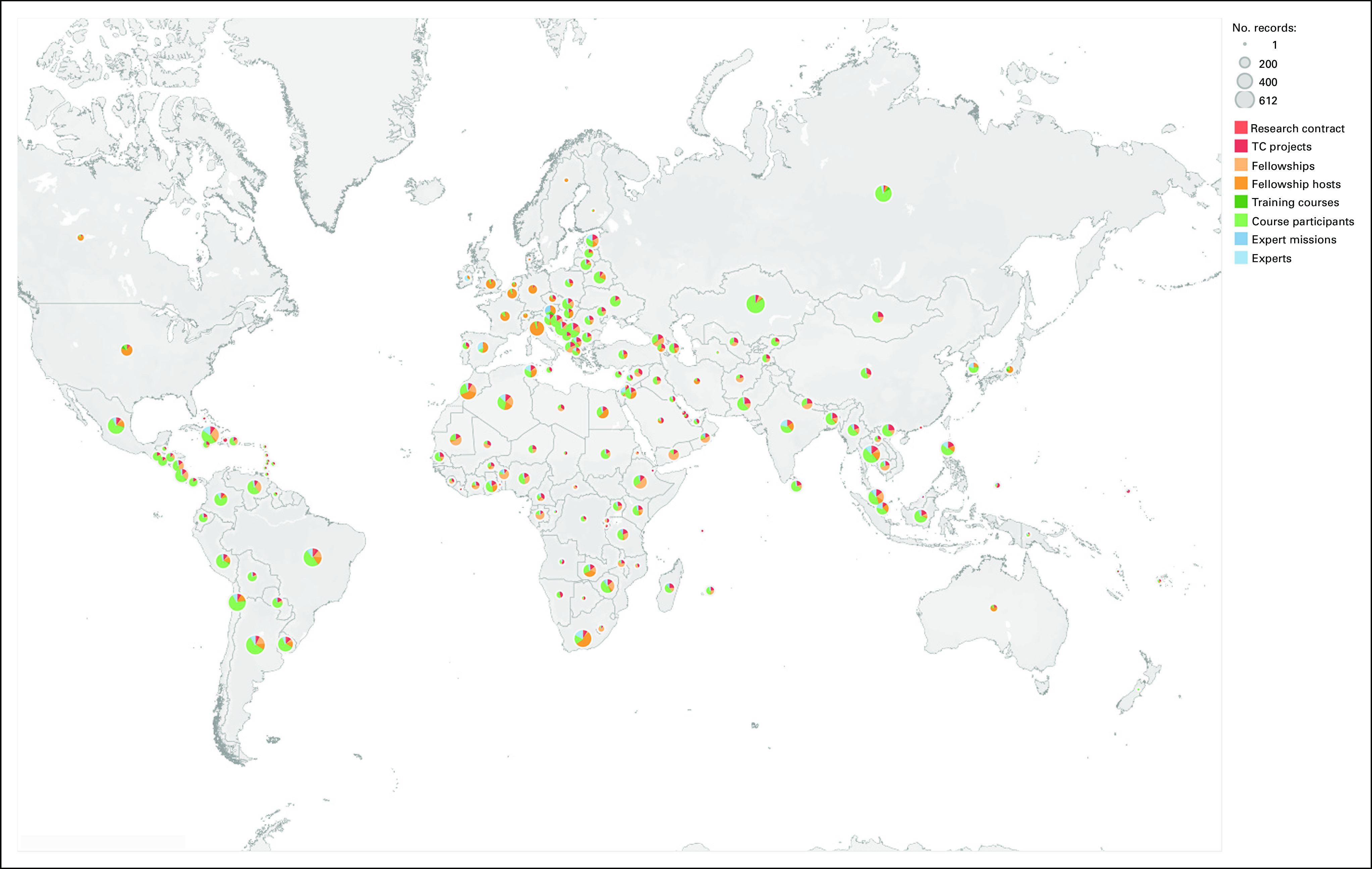
Coordinated research activities and in-country training, fellowship activities and expert support supported by IAEA Division of Human Health (NAHU). Color shows details about EventTopic. Size shows sum of number of records. IAEA, International Atomic Energy Agency; TC, technical cooperation. © 2021 Mapbox © OpenStreetMap.

Directories and databases are essential for keeping track of the activities in global radiotherapy. Some directories like IAEA's Directory of Radiotherapy Centers^15^ already exist, but more efforts are needed in the future. Directories itemizing cross-border exchanges especially with training and re-training opportunities should be available globally. Also, examples of effective partnerships and twinning arrangements between institutions and organizations may be helpful to those who aim to introduce such programs. The IAEA is currently setting up a global database, to be hosted on the IAEA Human Health Campus, to improve communication and facilitate collaboration in this area (Fig 7).^51^

**FIG 8:**
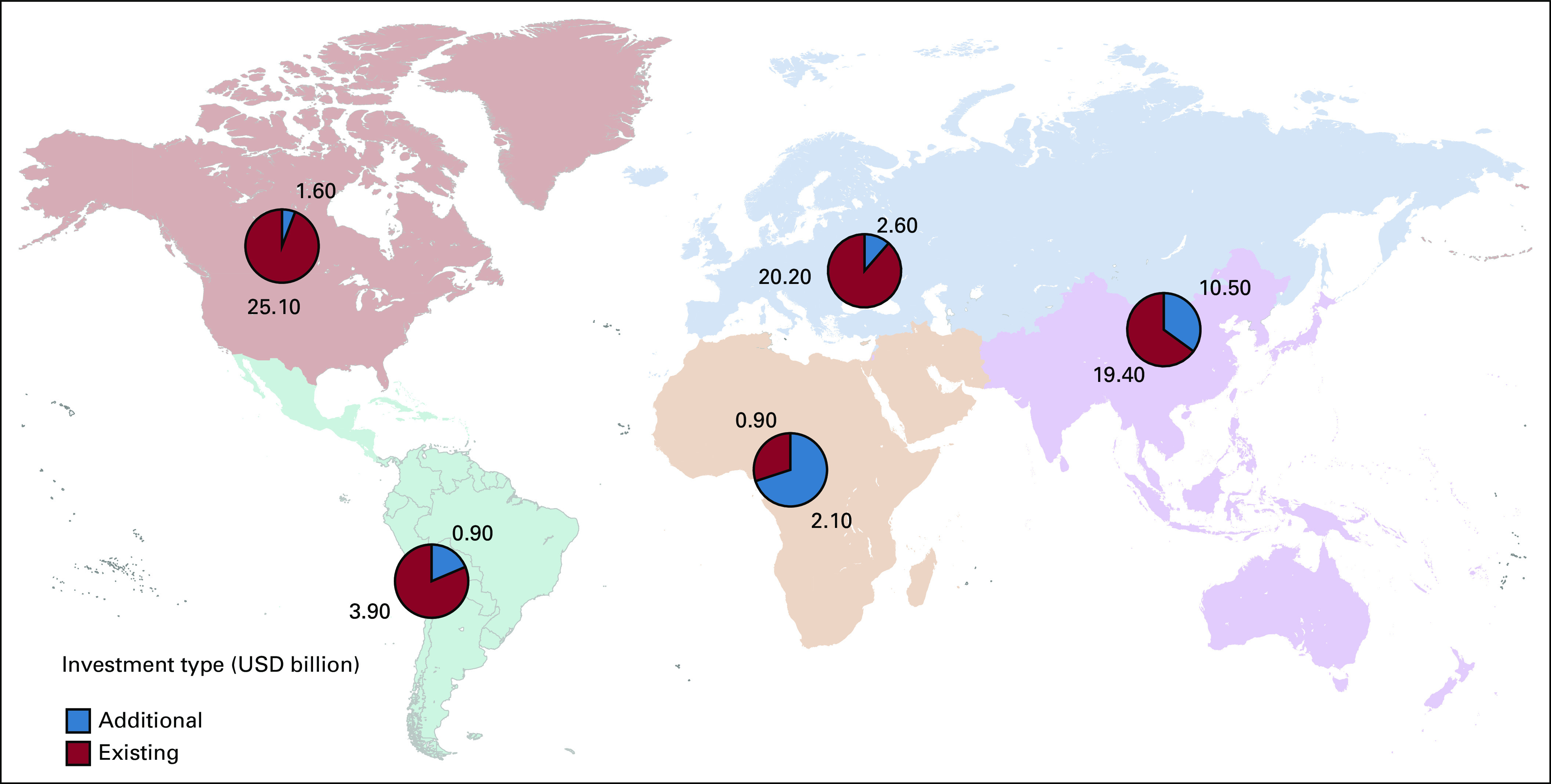
Current MV and additional needed MV machines. MV, megavoltage; USD, US dollars.

Collaborative, data-driven policymaking in radiotherapy should be integrated with other elements of the health system. Different organizations, projects, and collaborations have devoted efforts to producing evidence and developing tools supporting Health Services Research questions in the context of radiation oncology. Yet, the evidence on certain aspects—such as accurate resource costs and the value of radiotherapy—remains scarce and scattered to date. Moreover, the actual context of rapidly evolving radiotherapy treatments, techniques, and technologies, set within an equally fast changing oncology landscape, renders available evidence quickly outdated. This calls for accurate prediction models and continuous re-appraisal of availability and access, cost-effectiveness and value, and acceptability and affordability of radiotherapy innovations for the wider society. This is an endeavor that cannot be tackled by individual organizations or projects but would benefit a broad collaboration of all actors in the field involved with Health Services Research in radiation oncology. Only by joining our efforts will the radiation oncology community be able to continuously provide the adequate information necessary to foster radiotherapy at local, national, and global level, to the benefit of all patients with cancer who need radiotherapy as part of their multidisciplinary treatment (Fig 8).

Training and education of professionals in radiotherapy is at the center of sustainable delivery of services. Innovative approaches and harmonized curricula will be needed in the future. The IAEA has developed a master global curriculum for radiation oncologists, medical physicists, and radiation therapists. The IAEA and ESTRO are currently working together to produce a new updated curriculum in radiation oncology. To support education around the globe, curated content should be included in online platforms for easy access. Massive open online courses may provide much needed tools similar to IAEA courses through the IAEA Learning Management system such as Distance Assisted Training Online and The Applied Sciences of Oncology distance-learning course^52^ and others.^53^ Professional societies and other partners should leverage on the existing contents and materials to produce innovative tools and platforms for the next generation of professionals working in radiotherapy.

Modern information and communication technologies allow for faster implementation of modern technologies into clinical practice. Telecommunication, automation, remote support, virtual collaborative spaces, and partnerships are all underutilized in health care and should accelerate cost-effective practice. The IAEA has moved forward with technology advancements through virtual tumor boards such as Africa Radiation Oncology Network and distance learning through Human Health Campus, apps, and live streaming of conferences and seminars.^54,55,56,57^ Technology innovation will help to close the gap in access to radiotherapy. Implementation of new radiotherapy services should always be combined with efforts to expand education and research.

Innovative approaches for training have been envisaged. Because of its workplace-based nature, the education and training of radiotherapy professionals will require a significant proportion of it spent locally or at least in environments similar to the expected environments in which graduates will work in.^58^. However, this does not necessarily mean that professional education has to be organized in discrete, highly controlled silos. Throughout the longitudinal process of training and assessments, there exist various opportunities for a trainee to gain access to experts and other relevant resources at a global level—the only issue is to make this connection readily visible by the trainee. A central hub offering a main function as a global portfolio documenting training and assessments of trainees will allow a rudimentary structure for a global training program, and by connecting international initiatives with various training and assessment requirements, a blended learning environment will be created in which the local training experience can be enriched and enhanced by interaction with experts, resources, and fellow trainees at the global level. One example of such an approach is IAEA's AMPLE (Advanced Medical Physics Learning Environment) platform.^59^ This IAEA-developed environment provides residents medical physicists with guided learning materials and remote mentorships to enhance their clinical training in hospitals.

This global portfolio will also promote mutual recognition on a global level, supporting further exchanges occurring at the offline level and building strong ties between future professionals at a very early level of their careers. Medical education is a lifelong process that extends beyond medical schools and residency programs, well into the domains of continuous professional development.^60^ Although it is currently globally very challenging to have any measure of control over how knowledge, skills, and attitude develop beyond formal education and training, the portfolio can also allow practicing professionals to keep abreast with the evolving standards in the profession.

The IAEA will play a strategic role in the development of this initiative. Being an independent intergovernmental organization in the UN family, the IAEA can potentially create bridges over barriers faced by national or regional professional organizations. The series of syllabi published for radiation oncologists, medical physicists, radiation therapists, and oncology nurses are global standards, and such efforts can be achieved through collaboration with various societies and professionals under one umbrella. In addition, the IAEA's human health campus has seen sustained 20% growth annually in the number of users from 210 countries and territories that access its e-learning modules, webinars, and other resources. Since its official launch in 2010, it has shown great promise in its use as a sustainable platform for the education and training of professionals in the field of radiation medicine.^51^ The IAEA's mandate places it in a strategic role in supporting the education and training of radiotherapy professionals on a global level, and further development of existing resources such as the Human Health Campus would be beneficial to support the development of a global collaborative approach in the education and training of radiotherapy professionals.

Global competency–based credentialing, certification, and accreditation of training and education programs remain a challenge in global radiotherapy. Coordinated efforts among the various partners involved are needed to offer health professionals the highest standards to develop their professional careers.

The global future of radiotherapy will require us to focus on quality, safety, continuing education, access to treatment, advocacy, and sustainability. To ensure that initiatives are targeted to that effect, efforts and resources must be combined and coordinated by global independent organizations such as the IAEA, for example, in partnership with other major stakeholders in the field, such as major professional organizations. At the core should be the reinforcement of the value of radiotherapy, improving communication among the stakeholders worldwide, organizing and boosting the already ongoing work, and avoiding repetition where initiatives already exist.

Technical advances in radiation oncology, including hardware (image-based dose delivery systems) and software (evaluation tools and metrics integrated in treatment planning systems), have allowed dose escalation and treatment intensification in a very precise way. Although precision is technology-driven, outcome is ultimately dependent on biology. Modern imaging includes metabolic, biochemical, physiologic, and functional categories and can be linked to molecular and genetic profiling of both tumor and normal tissue, as well as novel therapeutic schedules. This process is often called molecular target profiling and can be integrated with increasing technical precision of radiotherapy. Biologic target delineation based on fluorodeoxyglucose positron emission tomography-computed tomography treatment planning is routine practice at present in many departments, but new tracers and other imaging modalities like positron emission tomography-magnetic resonance imaging will define new signatures and molecular targets for treatment.^61,62^

### New Directions

Personalized medicine builds on several research areas: medical imaging; biomarkers; molecular, genotypic, and environmental data; and tissue microarray for genetic profiling, among others. Linking the output of these research areas with historical clinical data can lead to personalized diagnosis and optimal treatment for both antitumor and side effects.^63^ It is now accepted that improved biologic profiling of individual patients is needed to better link patients with treatment schedules combining biologically targeted therapies with radiotherapy. Radiogenomics and pharmacogenomics will be increasingly important for prediction of efficacy as well as acute and long-term side effects. Of note is the complexity of this approach, where multiple signaling pathways and targets interact with the ionizing radiation on both tumor and normal tissues. This complexity necessitates a systematic research approach that includes radiotherapy to achieve optimal results. A new design of clinical trials will also be needed, going beyond traditional inclusion criteria and rigid designs to more adaptive^64^ and pragmatic studies.^65^

Spatially fractionated radiation therapy (SFRT) intentionally delivers a nonuniform dose distribution to the gross tumor volume, and it represents a crossover from the laboratory to the clinic.^66^ The radiobiologic rationale for SFRT is built on hypotheses and research suggesting specific molecular and cellular bystander mechanisms, alteration of the endothelium, and the interaction with the systemic immune system.^67,68^ SFRT is a novel technique, but early clinical results in bulky or locally advanced tumors demonstrate good response rates, prompting the development of controlled clinical trials. Several techniques are described to deliver SFRT (GRID, LATTICE, microbeams, and proton arrays), but a more conceptual development of the technique is needed, as well as further research to better understand the indications of the technique and the effects on tumor and normal tissues.

Nanomedicine is another active area of research in radiation oncology. Theranostics was originally coined as a term to describe a treatment modality combining a diagnostic test with targeted therapy based on the test results.^69^ Recent research will permit integration of nanotechnology into a theranostic platform, which can diagnose, deliver targeted therapy (including nanoradiotherapy and nanothermotherapy), and monitor the response to therapy.^70^ Various nanostructures have been described with a potential role in this new theranostic such as thermomagnetic nanotechnology for hyperthermia. Leveraging the interplay between nanomedicine, cancer biology research and technology, and clinical applications may start to emerge.^71^

In conclusion, the landscape of the global health community is now quite crowded with multiple actors including UN agencies, development assistance agencies, global health communities, academia, professional societies, nongovernmental organizations, patient support groups, and others. Although generally more is better, fragmentation of effort is not. It is important to communicate, coordinate efforts, collaborate with partners, and avoid the silo mentality.

Countries and regions across the globe are diverse in national income, funding for medical research and development, availability and type of health care system, governmental support, policies and national priorities, human resources availability, health infrastructure, treatment accessibility, and cancer registry with regional epidemiologic specificities. It is very important to identify the gaps in needs and resources and to harmonize and support education and training of personnel, taking into account the specificities of the country or region.

Regardless of all the efforts made to date, progress has been slow. There are indeed many challenges in improving cancer control and access to radiotherapy. Financing is frequently mentioned as the main barrier to radiotherapy in LMICs. Even if investing in facilities and infrastructure may seem to be the most important hurdle, training health professionals to provide a safe and effective treatment, including radiotherapy, presents a huge challenge considering the long time to competency. Building facilities and introducing safe and efficient practices are equally important.

Radiotherapy is now an established important component in cancer management; however, access to this treatment modality remains limited in many countries. Several organizations and professional bodies are working together to improve access to radiotherapy by demonstrating its cost effectiveness, providing toolkits for its advocacy, and supporting its safe and efficient utilization. Despite all these efforts, much remains to be done to advance radiotherapy and include this treatment modality in national cancer control programs with an acceptable level of access, quality, and safety worldwide.

The global future of radiotherapy will require us to focus on continuing education, management and access to treatment, safety regulations, health technology assessment, advocacy, and sustainability. To ensure that all initiatives are targeted to that effect, efforts and resources must be combined and coordinated at the international level. At the core should be the reinforcement of the value of radiotherapy, improving communication among the stakeholders worldwide, organizing and boosting the already ongoing work, and avoiding repetition where initiatives already exist. Effective collaborations on a regional and global level should be pursued, as it allows more rapid and efficient exchanges in expertise. This leads to improvements in the productivity and quality of education systems in producing trained professionals.
